# The first sixty days of COVID-19 in a humanitarian response setting: a descriptive epidemiological analysis of the outbreak in South Sudan

**DOI:** 10.11604/pamj.2020.37.384.27486

**Published:** 2020-12-30

**Authors:** Joy Luba Lomole Waya, Richard Lako, Sudhir Bunga, Helen Chun, Valerie Mize, Boniface Ambani, Joseph Francis Wamala, Argata Guracha Guyo, John Henry Gray, Malick Gai, Sylvester Maleghemi, Matthew Kol, John Rumunu, Michael Tukuru, Olushayo Oluseun Olu

**Affiliations:** 1 COVID-19 Response Team, World Health Organization, Juba, Republic of South Sudan,; 2 National COVID-19 Incident Management System, Ministry of Health, Juba, Republic of South Sudan,; 3 Centers for Disease Control and Prevention, Atlanta, Georgia, United States of America,; 4 National Public Health Emergency Operations Centre, Juba, Republic of South Sudan,; 5 Ministry of Health, Directorate of Preventive Health Services, Juba, Republic of South Sudan

**Keywords:** Novel coronavirus disease, severe acute respiratory syndrome coronavirus 2, epidemiology, humanitarian response setting, South Sudan, Africa

## Abstract

**Introduction:**

the coronavirus disease 2019 (COVID-19) was declared a pandemic on March 11, 2020. South Sudan, a low-income and humanitarian response setting, reported its first case of COVID-19 on April 5, 2020. We describe the socio-demographic and epidemiologic characteristics of COVID-19 cases in this setting.

**Methods:**

we conducted a cross-sectional descriptive analysis of data for 1,330 confirmed COVID-19 cases from the first 60 days of the outbreak.

**Results:**

among the 1,330 confirmed cases, the mean age was 37.1 years, 77% were male, 17% were symptomatic with 95% categorized as mild, and the case fatality rate was 1.1%. Only 24.7% of cases were detected through alerts and sentinel site surveillance, with 95% of the cases reported from the capital, Juba. Epidemic doubling time averaged 9.8 days (95% confidence interval [CI] 7.7 - 13.4), with an attack rate of 11.5 per 100,000 population. Test positivity rate was 18.2%, with test rate per 100,000 population of 53 and mean test turn-around time of 9 days. The case to contact ratio was 1: 2.2.

**Conclusion:**

this 2-month initial period of COVID-19 in South Sudan demonstrated mostly young adults and men affected, with most cases reported as asymptomatic. Systems´ limitations highlighted included a small proportion of cases detected through surveillance, low testing rates, low contact elicitation, and long collection to test turn-around times limiting the country´s ability to effectively respond to the outbreak. A multi-pronged response including greater access to testing, scale-up of surveillance, contact tracing and community engagement, among other interventions are needed to improve the COVID-19 response in this setting.

## Introduction

The global spread of the coronavirus disease 2019 (COVID-19) caused by the novel severe acute respiratory syndrome coronavirus 2 (SARS-CoV-2) is unprecedented. The disease attained the status of a pandemic on March 11, 2020 [1]. As of October 25, 2020, there were 43,341,451 globally confirmed cases and 1,157,509 deaths. The African continent continues to account for less than 5% of global confirmed cases and reported deaths, despite the continent being home to 17% of the global population [2]. South Sudan confirmed its first case of COVID-19 on April 5, 2020. Lockdown measures, including school closures, restriction of mass gatherings and commercial flights, were implemented approximately three weeks before the confirmation of the first cases. A predictive model projected an exponential rise of cases in the country with a peak three to four months after identification of the first case [3]. Due to the complex humanitarian crisis context and weak health systems, South Sudan remains ill prepared to effectively respond to the epidemic.

Chronic underdevelopment due to prolonged civil war prior to independence on July 9, 2011, further exacerbated by major civil conflicts in 2013 and 2016, has left South Sudan with one of the weakest health systems in the world. The prolonged years of conflict have resulted in a complex humanitarian crisis leading to massive population displacement with an estimated 7.5 million people in need of humanitarian assistance. Approximately 2.3 million South Sudanese refugees are in neighboring countries and 1.6 million are internally displaced persons, 181,000 of whom live in protection of civilians sites [4] where the overcrowded living conditions and inadequate access to social services challenge effective COVID-19 prevention and control. The country´s overall availability and capacity of health services is severely limited with a core health workforce density of 7.6 per 10,000 population, inpatient bed capacity of 6.5 per 10,000 population, and no functional intensive care units [5]. This lack of capacity is further compounded by socio-economic indicators that pose a threat to the effective implementation of community-based COVID-19 prevention and control strategies, including high poverty rates [6], poor living conditions, large household sizes [7], inadequate access to water and sanitation [8], and low literacy rates [9] among others.

Lower reported rates of COVID-19 morbidity and mortality in Africa to date have been attributed to a number of potential factors including the socio-ecological context, demographic differences, chronic disease patterns, early implementation of containment measures, and low COVID-19 diagnostic capacity [10, 11]. Compared with Europe, the Americas and Asia, weak health system capacity in most African countries, characterized by limited health infrastructure, diagnostic capacity, human resources for health, and insufficient funding for the health sector, is expected to be a determining factor in its ability to respond to cases and prevent further transmission [12]. The objective of this analysis is to describe the epidemiologic and socio-demographic characteristics of COVID-19 in South Sudan to inform ongoing prevention and control efforts, contain transmission levels and minimize damage caused by the pandemic. We also aim to contribute to the global knowledge on the epidemiology of COVID-19 in humanitarian response and low-income settings.

## Methods

### Study design

We conducted a cross-sectional descriptive epidemiological analysis of data collected through the South Sudan Ministry of Health (MoH) national COVID-19 surveillance system for the period from April 5, 2020 through June 5, 2020. This system was established in March 2020 by the MoH as part of its preparedness and response to COVID-19 using the WHO case definitions for surveillance [13]. The surveillance system receives alerts of suspected COVID-19 cases from the community, health facilities and points of entry (POE), transmitted via a toll-free hotline (6666) to the Public Health Emergency Operations Centre (PHEOC). At the sub-national level, state and county surveillance officers receive and verify alerts. Alerts that meet the case definition are investigated by a Rapid Response Team (RRT), comprised of a clinician, surveillance officer, laboratory technician, infection prevention and control officer, and data clerk. RRT deployment for case investigation utilizes a case investigation form that captures detailed socio-demographic, epidemiologic, and clinical data. Contact tracers utilize a contact tracing form that captures detailed epidemiologic data from contacts. Respiratory samples from suspected COVID-19 cases are collected using a nasopharyngeal or oropharyngeal swab and tested at the National Public Health Laboratory for COVID-19 using a WHO/FDA approved reverse transcription polymerase chain reaction (RT-PCR) diagnostic panel. Following laboratory confirmation of COVID-19 in suspected cases, cases are isolated and managed according to national guidelines, with contact elicitation, listing, tracing and follow-up carried out by contact tracing teams. The case investigation form is updated with test results, and cases and contacts are linked using unique identification. In addition to investigation and testing of verified alerts, samples for COVID-19 testing are also obtained from Severe Acute Respiratory Illness/Influenza-Like Illnesses sentinel surveillance sites, from screening of travelers at POE, and during pre-travel screening for those travelling from Juba to other locations within the country. The details of all COVID-19 alerts, cases, contacts, and laboratory tests are entered into their respective databases at the PHEOC.

### Data collection and analysis

We extracted, cleaned and conducted descriptive epidemiological analyses of the datasets from the COVID-19 databases for alerts, cases, contacts, and laboratory tests from April 5, 2020 through June 5, 2020. Cases were stratified by suspected case source and analyzed by age, sex, nationality, occupation, travel history, and geographic distribution; an epidemiologic curve was generated. Disease severity as per the national case management guidelines for COVID-19 [14], symptom range, proportion of cases admitted to a health facility, recovered, or died were analyzed. The case to contact ratio, proportion of contacts who became cases and the contact follow-up rate were calculated. To assess the performance of the surveillance and laboratory systems, the turn-around time (TAT) for alert investigation, sample positivity rate, tests per 100,000 population, and average time in days from sample receipt to results release by the PHEOC (RT-PCR test TAT) were calculated. The case fatality rate (CFR), epidemic doubling time, and attack rate were calculated. All datasets were analyzed using Microsoft Excel (version 2016) statistical tools.

## Results

### Socio-demographic and epidemiological characteristics among COVID-19 cases

**Figure 1:**
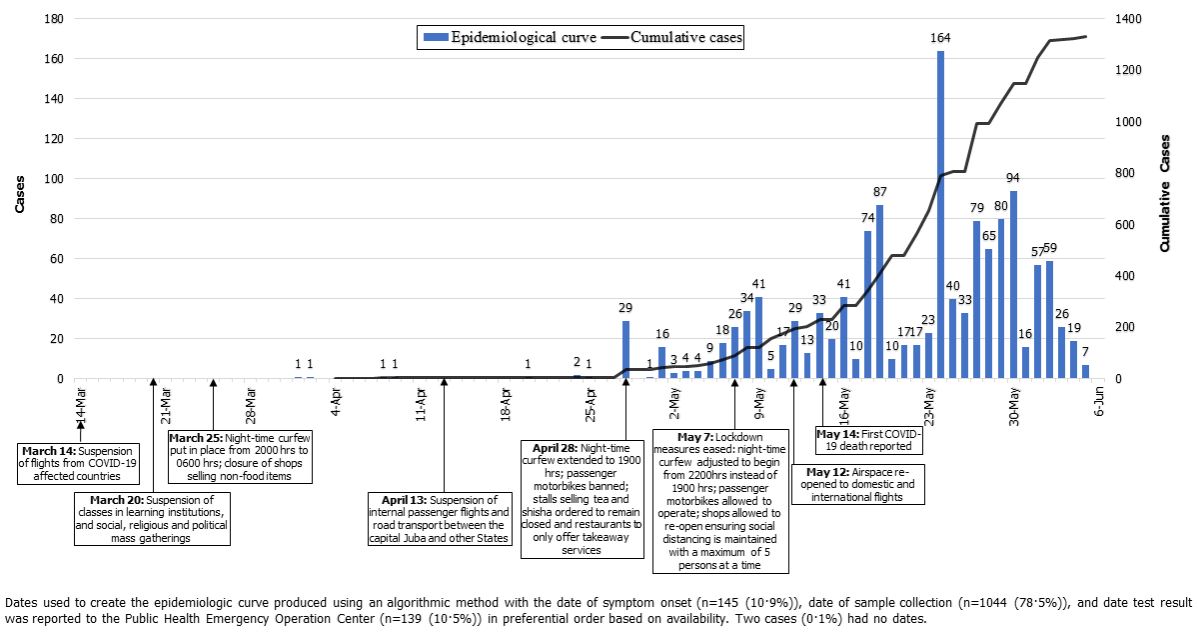
epidemiological curve and timeline of response events for COVID-19 in South Sudan

**Figure 2:**
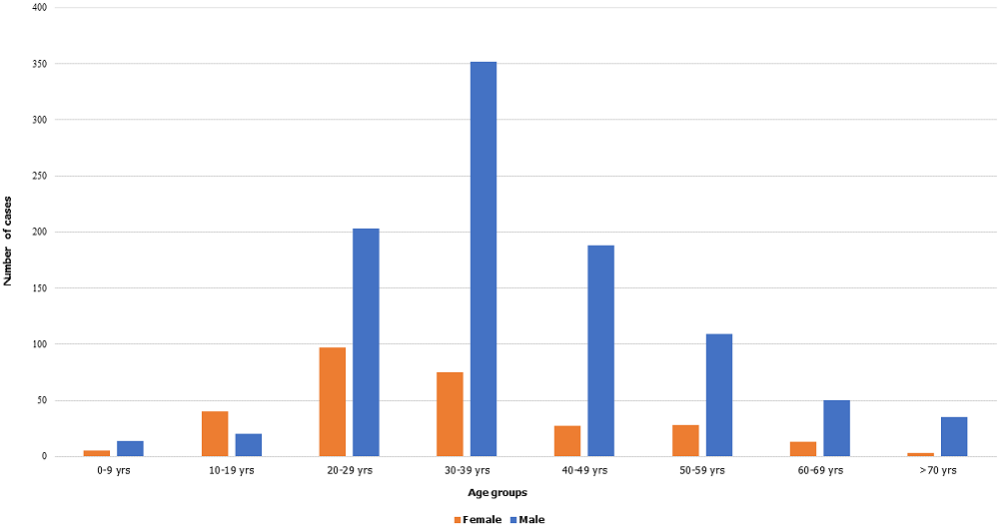
age and sex distribution of confirmed COVID-19 cases in South Sudan, April 5 to June 5, 2020 (n=1259)

From April 5, 2020 through June 5, 2020, South Sudan reported 1,330 confirmed cases of COVID-19 (Figure 1) with an attack rate of 11.5 per 100,000 population. The epidemic doubling time was on average 9.8 days (95% confidence interval [CI] 7.7 - 13.4). At the end of the 60-day period, six cases (0.5%) recovered and fourteen cases died, CFR of 1.1%. Eleven cases (0.8%) were isolated or admitted to a designated COVID-19 health facility, with the remainder managed from home (Table 1). Only 226 cases (16.9%) were symptomatic; of these, the most common symptoms were fever and chills (48.9%), rhinitis (45.0%), headache (39.3%), and malaise (34.1%). Of the 102 symptomatic cases whose disease severity was characterized, most (95%) were mild, one case (1%) was moderate, three cases (3%) were severe, and one case (1%) was critical. Seventy-five percent of cases were between the ages of 20 - 49 years, with a mean age of 37.1 years (Figure 2). The mean and median age of death, however, was 66.5 years (range 59.5 - 73.8). Males accounted for 77.0% of all cases, 92.9% of all deaths, and 81.8% of all individuals tested. Females were more likely to be tested through sentinel surveillance sites and contact tracing than through alerts or traveler screening (Table 2).

**Table 1:** socio-demographic and epidemiological characteristics of COVID-19 cases in South Sudan, April 5 to June 5, 2020

Variable	Number (n)	Percentage (%)/Rate
**Tests conducted**	7,326	53 per 100,000 population
**Cases**	1,330	
**Sample positivity rate (%)**		18.2%
**Epidemic doubling time (days)**	9.8 (95% CI 7.7 - 13.4)	
**Cumulative attack rate**	1,330	11.5 per 100,000 population
**Case fatality rate (%)**	14	1.1%
**Severity of case (n=102)**		
Mild	97	95%
Moderate	1	1%
Severe	3	3%
Critical	1	1%
**Hospitalized cases**	11	0.8%
**Recovered cases**	6	0.5%
**Occupation (n=576)**		
Professionals including international organizations workers	181	31.4%
Trader/businessperson	36	6.3%
Student	47	8.2%
Housewife	39	6.8%
Healthcare worker	41	7.1%
Security/traffic officer	89	15.5%
Government/civil servant	59	10.2%
Driver	68	11.8%
Farmer/pastoralist	10	1.7%
Other	6	1.0%
**Nationality (n= 1285)**		
South Sudanese	1,205	93.8%
Others (Kenyan, Ugandan, Congolese, Eritrean)	80	6.2%
**Imported cases**	20	1.5%
**Travel history (n=1330)**		
Yes	23	1.7%
No	1,307	98.3%
**Source of detection of cases (n=1308)**		
Alerts	164	12.5%
Contacts of cases	389	29.7%
Sentinel sites	159	12.2%
Travel screening	596	45.6%
**Turn Around Time (TAT) for alert investigation (hours) (n= 220)**		
< 24 hours	186	84.5%
48 hours	4	1.8%
> 48 hours	30	13.6%
**Reverse Transcription Polymerase Chain Reaction (RT-PCR) TAT (Average days)**	9	

**Table 2:** persons tested, cases, source of case detection and deaths by sex in South Sudan, April 5 to June 5, 2020

Variable	Number (%)	
**Male**	**Female**
Persons tested (n=3,750)	3,068 (81.8%)	682 (18.2%)
Cases* (n=1,320)	1,016 (77.0%)	304 (23.0%)
Cases detected through traveler screening (n=596)	484 (81.2%)	112 (18.8%)
Cases detected through the alert system (n=164)	130 (79.3%)	34 (20.7%)
Cases* detected as contacts of cases (n=380)	291 (76.6%)	89 (23.4%)
Cases* detected through sentinel surveillance (n=158)	104 (65.8%)	54 (34.2%)
Deaths (n=14)	13 (92.9%)	1 (7.1%)

* Cases include data where sex disaggregation was available.

**Figure 3:**
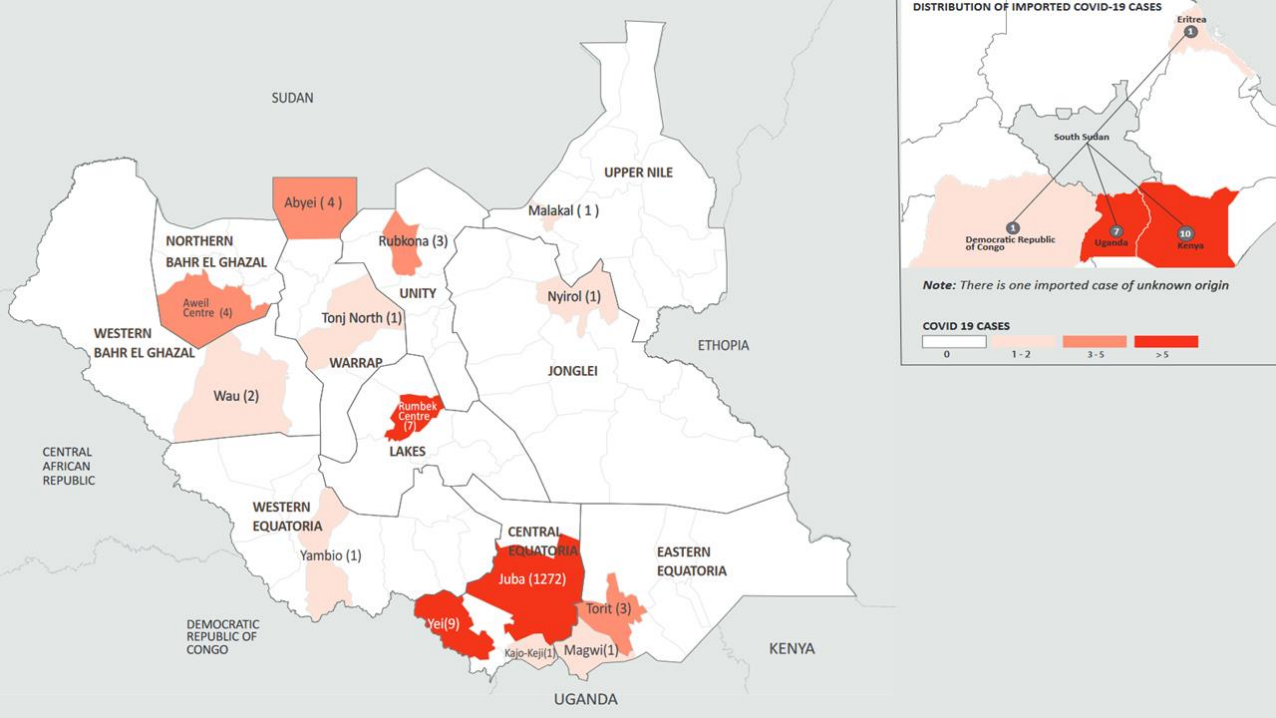
geographic distribution of confirmed COVID-19 cases by county in South Sudan, April 5 to June 5, 2020

Forty-six percent of cases were detected through traveler screening, including testing of truck drivers at POE and in-country travelers departing from the capital city, Juba. A substantial percentage of cases were also detected through contact tracing (29.7%), while alerts and sentinel site testing accounted for 12.5% and 12.2% of all cases detected, respectively (Table 1). The highest prevalence and incidence of cases was from the capital city, Juba, with an incidence of 312.3 cases per 100,000 population (Table 3, Figure 3). Most confirmed cases (98.3%) reported no history of travel out of the country, and 1,205 of 1285 cases (93.8%) were South Sudanese nationals. Analysis of occupation of confirmed cases showed one third were professionals, including international non-governmental organization workers, while healthcare workers (HCWs) accounted for 7.1% (41 of 576) of cases (Table 1).

**Table 3:** cumulative cases of COVID-19 by county in South Sudan

County/Administrative Area	State	Population Estimate*	Cases	Incidence per 100,000 population
Juba	Central Equatoria	407,323	1,272	312.3
Yei	Central Equatoria	167,550	9	5.4
Rumbek Centre	Lakes	269,537	7	2.6
Abyei Administrative Region	N/A	N/A	4	N/A
Aweil Centre	Northern Bahr el Ghazal	114,099	4	3.5
Rubkona	Unity	77,683	3	3.9
Torit	Eastern Equatoria	160,818	3	1.9
Wau	Western Bahr el Ghazal	296,384	2	0.7
Malakal	Upper Nile	76,907	1	1.3
Kajo-keji	Central Equatoria	109,771	1	0.9
Nyirol	Jonglei	181,159	1	0.6
Magwi	Eastern Equatoria	192,003	1	0.5
Yambio	Western Equatoria	201,199	1	0.5
Tonj North	Warrap	249,779	1	0.4

* Population estimates from 2019 (https://data.humdata.org/dataset/south-sudan-population-statistics).

### Socio-demographic and epidemiological characteristics among health care workers

The attack rate among HCWs was 520 per 1000, with demographics similar to those of overall cases (mean age of 38.6 years, 73.2% males, and 26.8% females). One of the 41 infected health workers died, giving a CFR of 2.4%.

### Contact tracing and laboratory performance

For the 1,330 cases, only 813 cases (61.1%) had contacts listed, and only 62 of the cases had five or more contacts. A total of 2,920 contacts were listed for a case to contact ratio of 1: 2.2. Of the contacts, 389 (13.3%) were later confirmed to be cases. Less than one third (29.2%) of cases were listed as contacts prior to infection. Among the 7,326 RT-PCR tests conducted, the proportion positive was 18.2%, with a TAT averaging 9 days. COVID-19 test rate per capita was 53 per 100,000 population.

## Discussion

Our analysis from the first two months of the COVID-19 outbreak in South Sudan provides insights that inform prevention and control policies, strategies, and activities, and contributes to the global knowledge on the epidemiology of COVID-19 in a humanitarian response and low-income setting. The majority of cases observed were asymptomatic (83%), higher than the range of 4% to 80% reported globally [15]. In other studies, a proportion of initially asymptomatic individuals went on to develop symptoms, while others remained asymptomatic [16]. Data regarding initially asymptomatic cases who later developed symptoms in South Sudan were limited, and determination of possible reasons for a higher proportion of asymptomatic cases in this context is needed. The range of symptoms in South Sudan is similar to what has been reported in Africa and elsewhere [17, 18]. Although South Sudan´s initial testing scheme included testing individuals with symptoms of potential COVID-19, samples collected from sentinel surveillance sites, travelers (arrivals and within country) including truck drivers crossing into South Sudan from neighboring countries, and high-risk contacts regardless of symptoms, adherence to the recommended criteria-based prioritization was not verified. Discussions of testing prioritization and optimization among select populations and settings are ongoing using available data to inform recommendations.

Although SARS-CoV-2 prevalence has been reported as similar between the sexes or marginally higher in males than females [19, 20], over 75% of cases in this analysis were male. This trend may be attributed to more males being tested than females, given men travel more and most truck drivers are male; highlighting a continued need for greater test per capita coverage. Risk factors for men becoming infected include behaviors such as congregating in crowded settings (e.g., drinking houses), nonadherence to public health measures such as hand washing, or having higher rates of underlying chronic conditions; the validity of these potential risk factors needs to be further explored [21]. Our findings suggest a higher risk of death among males, similar to what has been observed in other countries [22]. Young adults between 20 and 49 years bear the burden of COVID-19 in South Sudan, similar to patterns seen in most African countries [23]. COVID-19 deaths, however, are disproportionately higher in older age groups, and given that 59% of the population in Africa is below the age of 24, fatalities are predicted to be lower there than in other regions [24, 25]. There were four times more HCWs infected than COVID-19 cases isolated or admitted to a health facility, suggesting HCWs are getting infected in the community. Studies on the transmission dynamics and risk factors of COVID-19 among HCWs are needed. Less than 25% of cases were identified through ongoing surveillance such as alert and sentinel sites surveillance, suggesting limitations of the community and facility-based surveillance system with respect to coverage and yield and highlighting the improvements needed in this area. Despite the implementation of lockdown measures almost three weeks before the confirmation of the first cases, mitigation strategies such as physical distancing, masking, and adaptation of services to limit movement and contact among individuals were not frequently observed. Additionally, lockdown measures were lifted early in the course of the outbreak, and may have negatively influenced the epidemic curve, however, the limited duration and varying degrees of lockdown implementation limit the extent to which this attribution can be made.

The epidemic doubling time is similar to what has been observed in other countries in the initial phase of an outbreak [26], and in countries in the African Region. However, some studies estimate much shorter doubling times. Longer doubling times have been attributed to underreporting of cases, especially in contexts where there is limited testing capacity [27]. Limited COVID-19 testing capacity led to long TAT and irregular daily case reporting. Contributors to the long TAT for laboratory results include a limited number of RT-PCR platforms dedicated to COVID-19, and inadequate human resources and supplies required for testing. Although similar to that observed across sub-Saharan Africa, the overall test rate per capita is low in South Sudan compared to higher income countries [28]. South Sudan´s sample positivity rate is among the highest in sub-Saharan Africa and is higher than the recommended rate of 3 - 12%, implying inadequate testing and poor case detection [29]. Understanding modes of transmission, disease incidence, and prevalence in South Sudan will require greater testing coverage, improved case detection, and contact tracing.

The large household size and communal living patterns in South Sudan pose significant challenges for contact tracing. Less than one third of cases were listed as contacts of cases prior to confirmation, implying ongoing community transmission and highlighting the inadequate case identification and contact tracing. Stigma has been observed for other infectious diseases in Africa [30] and may hamper contact tracing efforts for COVID-19 with refusal by some cases to list their contacts. Efforts to prevent and combat stigma and discrimination experienced not only by the individual who has COVID-19, but their caregivers, family, friends, and communities will require community engagement at all levels. Many individuals in South Sudan have experienced psychological trauma from prolonged and ongoing conflict, poverty, hunger and the burden of other infectious diseases. Those who recover from COVID-19 or finish quarantine may suffer from anxiety and fear of being “marked” by COVID-19. Understanding these potentially complex psychosocial dynamics surrounding COVID-19 is crucial. It is important for governments and communities to identify potential strategies to prevent and limit any lasting psychological sequelae of COVID-19 and educate the community to eradicate behaviors that may hinder control of this disease.

## Limitations

This analysis has several limitations. COVID-19 databases were generally not linked or harmonized and had varying degrees of missing data for variables of interest including sex (0.8%), nationality (3.4%), age (5.3%), occupation (56.7%), and symptom categorization (54.9%). Individuals completing source forms have also been variably trained resulting in inconsistent data quality. The low proportion of contacts identified may also relate to poor data quality with multiple databases for contact tracing. We attempted to collect the missing information, where possible, and limited some analyses of case investigation data to those with complete data. Additionally, data on all deaths may not have been reported and may underestimate the true CFR.

## Conclusion

This is the first comprehensive epidemiological analysis of the characteristics of COVID-19 in South Sudan. Our data show the disease predominantly affecting younger adults and men, who seem to be accessing testing more than women. COVID-19 cases and deaths were lower than observed in other countries, although this may be due to the limitations in surveillance, testing and reporting. Furthermore, most of the COVID-19 cases were asymptomatic. Contact tracing performance was generally poor. Timely detection and testing of suspected cases, effective case isolation, and tracking and quarantining of contacts remain critical challenges to the outbreak response in South Sudan. We propose a number of recommendations based on our findings. First, greater COVID-19 testing capacity, quality and coverage are needed. Implementation of additional laboratory platforms and decentralized testing capacity should be considered with quality assurance for testing and training of additional laboratory personnel. TAT should continue to be monitored to ensure timely availability and use of results by the RRT, contact tracing teams, and for surveillance purposes. Second, COVID-19 surveillance and contact tracing should be strengthened across community and health facility levels using existing community health structures where possible, to improve the alert of suspected cases, listing of contacts, completion rates of cases in isolation and contacts in quarantine, and efficacy of risk communication. More contact tracers should be recruited and trained, and innovative digital health tools should be deployed to improve contact tracing timeliness, data capture quantity, quality and utilization for intervention impact. Investments in strengthening interoperable and harmonized data systems should be made to improve COVID-19 data management and other disease surveillance. Third, in view of the high percentage of asymptomatic cases observed, greater investments should be made to scale up home-based care, in line with the national case management strategy, to reserve critical resources for more severely affected cases. Fourth, a gender-sensitive approach to the outbreak response, especially in surveillance and testing, is required. Lastly, further studies are needed to better understand the seroprevalence of the disease among the general population and the transmission dynamics and risk factors among health care workers. The above recommendations and greater investments in behavior change communication and community engagement could provide South Sudan with the ability to confront the challenges posed by COVID-19.

### What is known about this topic

- COVID-19 prevalence has been reported as similar between the sexes or marginally higher in males than females;
- Weak health systems impede a country´s ability to effectively respond to the COVID-19 pandemic.

### What this study adds

- In settings with limited health system capacity, inequities in access to critical public health services such as COVID-19 testing, management and prevention may contribute to marked differences in the observed epidemiology of COVID-19 in these settings.
